# Proteoglycan 4 (Lubricin) and regulation of xanthine oxidase in synovial macrophage as a mechanism of controlling synovitis

**DOI:** 10.1186/s13075-024-03455-x

**Published:** 2024-12-19

**Authors:** Khaled A. Elsaid, Ling X. Zhang, Thomas Zhao, Ava Marks, Derek Jenkins, Tannin A. Schmidt, Gregory D. Jay

**Affiliations:** 1 Department of Biomedical and Pharmaceutical Sciences, Chapman University, Irvine, CA 92618 USA; 2 Department of Emergency Medicine, Rhode Island Hospital, Providence, RI USA; 3 Boston University, Boston, MA USA; 4 Brown University, Providence, RI USA; 5 Department of Orthopaedics, Rhode Island Hospital, Providence, RI USA; 6 Biomedical Engineering Department, School of Dental Medicine, University of Connecticut, Farmington, CT USA

**Keywords:** PRG4, Xanthine oxidase, Febuxostat, Glycolysis, Synovial macrophages

## Abstract

**Background:**

Synovial macrophages (SMs) are important effectors of joint health and disease. A novel Cx3CR1 + TREM2 + SM population expressing the tight junction protein claudin-5, was recently discovered in synovial lining. Ablation of these SMs was associated with onset of arthritis. Proteoglycan 4 (PRG4) is a mucinous glycoprotein that fulfills lubricating and homeostatic roles in the joint. The aim of this work is to study the role of PRG4 in modulating synovitis in the context of SM homeostasis and assess the contribution of xanthine oxidase (XO)-hypoxia inducible factor alpha (HIF-1a) axis to this regulation.

**Methods:**

We used *Prg4*^*FrtloxP/FrtloxP*^;*R26*^*FlpoER/+*^, a novel transgenic mouse, where the *Prg4*^*Frt*^ allele normally expresses the PRG4 protein and was designed to flank the first two exons of *Prg4* with a flippase recognition target and “LOXP” sites. Inducing flippase activity with tamoxifen (TAM) inactivates the *Frt* allele and thus creates a conditional knockout state. We studied anti-inflammatory SMs and XO by quantitative immunohistochemistry, isolated RNA and studied immune pathway activations by multiplexed assays and isolated SMs and studied PRG4 signaling dysfunction in relation to glycolytic switching due to pro-inflammatory activation. *Prg4* inactivated mice were treated with oral febuxostat, a specific XO inhibitor, and quantification of Cx3CR1 + TREM2 + SMs, XO immunostaining and synovitis assessment were conducted.

**Results:**

*Prg4* inactivation induced Cx3CR1 + TREM2 + SM loss (*p < 0.001*) and upregulated glycolysis and innate immune pathways in the synovium. In isolated SMs, *Xdh* (*p < 0.01*) and *Hif1a* (*p < 0.05*) were upregulated. Pro-inflammatory activation of SMs was evident by enhanced glycolytic flux and XO-generated reactive oxygen species (ROS). Febuxostat reduced glycolytic flux (*p < 0.001*) and HIF-1a levels (*p < 0.0001*) in SMs. Febuxostat also reduced systemic inflammation (*p < 0.001*), synovial hyperplasia (*p < 0.001*) and preserved Cx3CR1 + TREM2 + SMs (*p < 0.0001*) in synovia of *Prg4* inactivated mice.

**Conclusions:**

PRG4 is a biologically significant modulator of synovial homeostasis *via* inhibition of XO expression and downstream HIF-1a activation. PRG4 signaling is anti-inflammatory and promotes synovial homeostasis in chronic synovitis, where direct XO inhibition is potentially therapeutic in chronic synovitis.

**Supplementary Information:**

The online version contains supplementary material available at 10.1186/s13075-024-03455-x.

## Background

Proteoglycan 4 (PRG4)/lubricin is a mucinous glycoprotein secreted by synoviocytes and superficial zone chondrocytes [1, 2], with a unique role in the joint as a boundary lubricant and anti-inflammatory effectuator. PRG4 is configured as a polymer brush with a high adsorptive capacity on hydrophilic and hydrophobic surfaces [3, 4] where it forms a barrier nanofilm. The protein core is 1,404 amino acids long containing a heavily glycosylated central mucin domain, which prevents friction-induced mitochondrial dysregulation and chondrocyte apoptosis [5–7]. PRG4 also prevents synovial overgrowth and proliferation in response to mitogenic signals [8–10]. Loss of function mutations in *PRG4* are responsible for the autosomal recessive disease, Camptodactyly-Arthropathy-Coxa Vara Pericarditis (CACP) syndrome, a juvenile onset arthropathy [11]. This precocious arthropathy is characterized by protein biofouling and destruction of articular cartilage, and synovial hyperplasia which has not been fully characterized. As shown by us and others, *PRG4* expression by synoviocytes is reduced by IL-1β [9, 12]. PRG4 is also susceptible to proteolytic degradation by elastases and cathepsin G [13, 14], while truncation of its glycans promotes inflammation [15].

The synovium is a soft tissue that is comprised of a surface layer, the intima and an underlying subintima, with two cell types: synoviocytes and macrophages [16]. Chronic synovitis is characterized by synovial hyperplasia and recruitment of circulating monocytes which differentiate into macrophages. In addition, the synovium may be enriched in T cells and lesser extents mast cells, B cells and endothelial cells of newly formed blood vessels [17, 18]. In the *Prg4* null state, chronic synovitis is evident with accumulation of activated macrophages [19, 20] which contribute to progressive tissue remodeling. However, the mechanism through which PRG4 regulates synovial homeostasis is not completely understood.

Synovial macrophages (SMs) comprise a heterogenous group of functionally diverse populations that fulfill functions crucial to synovial homeostasis. Of interest is a distinct population of anti-inflammatory Cx3CR1 + TREM2 + SMs that form an immunological barrier in the intimal layer which in turn shields the synovium from the joint cavity and prevents activation of macrophages in the subintimal layer [21]. Disruption of this locally renewed layer of macrophages was associated with recruitment of circulating monocytes and onset of arthritis [21, 22]. Owing to its synovial localization and its bioactivity mediated by CD44 and toll-like receptor (TLR) interactions [23, 24], it is likely that erosion of the PRG4 layer results in barrier macrophage activation as PRG4 cloaks CD44 and TLR receptors and thus protects the anti-inflammatory phenotype of these SMs. CD44 is a single pass non-kinase transmembrane receptor that plays an important role in inflammation and shedding or internalization of the CD44 extracellular domain (as in the case with PRG4 binding) induces a conformational change in its intracellular domain that activates protein phosphatase 2 A (PP2A), which is anti-inflammatory *via* inhibition of NF-κB nuclear translocation [23, 25]. We posit that a dysregulation of the PRG4/CD44 axis in SMs is permissive for xanthine oxidase (XO) upregulation which generates reactive oxygen species (ROS) and activates the NLRP3 inflammasome [26, 27], hence shifting towards a pro-inflammatory phenotype. Ultimately this affects the extracellular matrix thus promoting synovial fibrosis in *Prg4* null and in posttraumatic OA (PTOA) animal models.

In this work, we studied early changes to the synovium, with respect to SM activation status as a result of failure of PRG4’s biological function, in a novel model of *Prg4* conditional inactivation. Using isolated SMs, we deciphered the molecular targets of PRG4’s function and identified XO and downstream hypoxia inducible factor alpha (HIF-1a) as the primary mechanism mediating SM activation. We also investigated whether febuxostat, a specific non-purine XO inhibitor [28], reverses synovial pathology in our *Prg4* conditional knockout mice.

## Methods

### Prg4 conditional inactivation and Prg4−/− mouse models

*Prg4*^*FrtloxP/FrtloxP*^;*R26*^*FlpoER/+*^ is a transgenic mouse, where the *Prg4*^*Frt*^ allele normally expresses the PRG4 protein and was designed to flank the first two exons of *Prg4* with a flippase recognition target and “LOXP” sites. Inducing flippase activity with tamoxifen (TAM) inactivates the *Frt* allele and thus creates a conditional knockout state. Tamoxifen (0.1 mg/gram in 100 μl corn oil; TAM) (Sigma Aldrich) or corn oil (Veh; 100 μl) (Sigma Aldrich) administrations occurred in 4 weeks-old animals for 10 days and histological analyses and synovial tissue collection for RNA and SM isolations were performed 6 weeks later. This model was created by Dr. Matthew Warman and Dr. Gregory Jay. Other *Prg4* mutant mouse models used was a global *Prg4* null mouse (*Prg4*^*−/−*^) and *Prg4* sufficient mouse (*Prg4*^*+/+*^) littermates from the breeding of *Prg4*^*+/−*^ mice [29]. All animal breeding and procedures were reviewed and approved by the Lifespan Institutional Review Board (IACUC of Rhode Island Hospital #504323).

### Histological analyses

Knee joints were harvested at 6 weeks following TAM or Veh administrations and subjected to decalcification, paraffin embedding and histological sectioning (*n* = 6 in each group with 3 males and 3 females). Histological sections (5 μm) were selected to include both meniscal horns as landmarks. Histological staining included PRG4 (Mouse ab S6.79 provided by Dr. Tom Schmidt [30]), Cx3CR1 (ab308613; recombinant anti-CX3CR1 rabbit antibody), TREM2 (ab305103; recombinant anti-TREM2 rabbit antibody), Claudin-5 (ab131259; recombinant anti-claudin 5 rabbit antibody) and XO (ab109235; recombinant anti-XO rabbit antibody) (Abcam) (1:100 dilutions for all antibodies) and incubated overnight at 4^o^C. Following washing with PBS, sections were incubated with HRP-goat anti-mouse IgG (ThermoFisher Scientific) (PRG4) or Cy3 goat anti-rabbit IgG (ThermoFisher Scientific) (Cx3CR1, TREM2, Claudin-5 and XO) at 1:200 dilution for 1 h at room temperature. Sections were washed with PBS and developed using DAB reagent (PRG4) or mounted in Vectashield mounting medium with DAPI (Vector Laboratories Inc.). Slides were imaged using fluorescence microscopy (Nikon, ECLIPSE E800) and quantified using Image J. Data are presented as fluorescence intensity measurements in TAM animals normalized to Veh animals. Sections were also stained with hematoxylin and eosin (H&E) and synovial pathology was assessed by two blinded investigators using a 0–3 score, where 0 = normal synovium, 1–2 cell layers thick and 3 = severe extensive hypertrophy > 5 cell layers and/or infiltration of synovium greater than 50% of surface [10].

### RNA isolation, cDNA synthesis, digital droplet PCR (ddPCR), qPCR and multiplexed gene expression panels

Mouse knee joints were harvested, and synovial tissues from both knee joints were collected, pooled and subsequently homogenized (*n* = 6 in each group with 3 males and 3 females). RNA was extracted and purified using RNeasy Mini kit (Qiagen). RNA concentration was measured by Nanodrop 2000c (ThermoFisher Scientific). High-capacity RNA-to cDNA kit (ThermoFisher Scientific) was used to generate cDNA from 1 μg of total RNA. TaqMan murine *Prg4* primer probe (ThermoFisher Scientific) was mixed with ddPCR supermix (Bio-Rad) and 50 to 100ng cDNA in 20 μl reaction volume. An automated generator (Bio-Rad) was used to generate droplets followed by PCR amplification. A ddPCR droplet counter machine QX 200 (Bio-Rad) was used for droplet reading, and results were quantified with Quantasoft software.

qPCR was performed as previously described [23]. Genes of interest included *Cd44* (Mm01277161_m1), *Xdh* (Mm00442110_m1), *Hif1a* (Mm00468869_m1), and *Gapdh* (Mm99999915_g1) as an internal control (ThermoFisher Scientific). Multiplexed gene expressions were performed using pre-validated panels for murine immune activation status and cell metabolism using the nCounter technology (NanoString). Data were analyzed using ROSALIND software and presented as directed global significance scores of different pathways and fold change of expression of target genes. Each group included 6 animals with 3 males and 3 females.

### Isolation of SMs and glycolytic activation studies

Synovial tissues from 10 Veh-administered animals (5 males and 5 female) and 10 TAM-administered animals (5 males and 5 females) were subjected to SM isolation and surface marker characterization [31, 32]. Glycolytic activation of cultured SMs (50,000 cells per well) was monitored in real-time using a Seahorse Analyzer (Seahorse XF HS Mini Analyzer; Agilent Technologies). Pro-inflammatory activation was performed using LPS (100 ng/mL) and IFNγ (20 ng/mL) ± drug treatments followed by OCR (oxygen Consumption Rate) and ECAR (Extracellular Acidification Rate) monitoring and calculation of PER (Proton Efflux Rate), which is an indicator of macrophage immune activation [33].

### ROS and HIF-1α quantifications in cultured SMs

Cultured SMs (500,000 cells per well) were activated using LPS (100ng/ml) and IFNγ (20ng/ml) ± drug treatments and ROS levels were quantified at 24 h using the DCFDA/H2DCFDA kit (Abcam). SMs were also collected following similar treatments and cellular HIF-1α levels were determined by ELISA (Abcam) and normalized to protein levels.

### In vitro pharmacologic treatments

SM treatments included rhPRG4 (200 μg/ml) (Lubris) [34], IM7 (15 μg/ml) (ThermoFisher), Isotype Control (IC; 15 μg/ml) (ThermoFisher), febuxostat (25μM) (Cayman Chemicals), N-acetylcysteine (NAC) (5μM) (Cayman Chemicals) and echinomycin (50nM) (Abcam).

### In vivo treatment with oral febuxostat

At 4 weeks old, mice received TAM or Veh as described above. Additionally, mice were treated with oral febuxostat (2.5 mg/kg/day) in drinking water for 6 weeks until euthanasia. Experimental groups included Veh (*n* = 5), TAM (*n* = 5), and TAM + Febuxostat (*n* = 5) (3 males and 2 females in each group). Febuxostat was dissolved in drinking water and the febuxostat solution was freshly made every 48 h. Sera were collected and serum IL-1β levels were measured by ELISA (Abcam). Peripherally circulating monocytes were isolated (MojoSort Mouse Monocyte Isolation Kit; Biolegend) and qPCR of target genes was performed as described above. Immunohistological staining and analyses were performed as described above.

### Isolation of CD14 + monocytes/macrophages from human OA synovial tissues and activation by LPS

Synovial tissues from three OA patients (2 males and 1 female; medial and lateral compartments) were cut into small pieces and incubated in 10 ml of DMEM containing 1 mg/ml of collagenase type 4 (220U/mg; Worthington Biochemical Corp) for 3 h at 37 °C. CD14 Dynabeads (ThermoFisher Scientific) were used to isolate CD14 + cells by magnetic separation using a DynaMag-15 magnet (ThermoFisher Scientific). Glycolytic activation (50,000 cells per well), using LPS (100 ng/ml), and rhPRG4 (200 μg/ml) treatment were performed as described above. Recovery and processing of human tissue for research purposes were authorized by the Lifespan Institutional Review Board (Human subjects review committee #412420) and written informed consent was obtained from participating donors.

### Statistical analyses

Statistical analyses of gene expression data were performed using ΔC_t_ values (C_t_ target gene - C_t_*Gapdh*), and data were graphically presented as fold change of TAM or TAM + febuxostat treatment compared with untreated littermate Veh controls. Directed global significance scores are similar to pathway scores in that the changes in expression levels of genes of a specific pathway are distilled into a principal component (significance score). These scores were scaled on the *t*-statistic, allowing for comparisons across groups and experiments. Directed global scores of higher magnitude (positive or negative) are indicative of stronger changes in the level of pathway gene expression. Positive scores indicate upregulation of the pathway, while negative scores indicate downregulation of the pathway. Similarly, gene expression data in TAM vs. Veh groups were analyzed using Student’s *t*-test. Statistical comparisons of multiple groups were performed using ANOVA for parametric data followed by *post* hoc Tukey’s test. A *p* value of 0.05 was considered statistically significant. Data are graphically represented as scatter plot bar graphs with mean ± S.D. indicated.

## Results

### Prg4 conditional inactivation results in synovitis, loss of barrier SMs and upregulation of innate immune and glycolysis pathways in the synovium

**Fig. 1:**
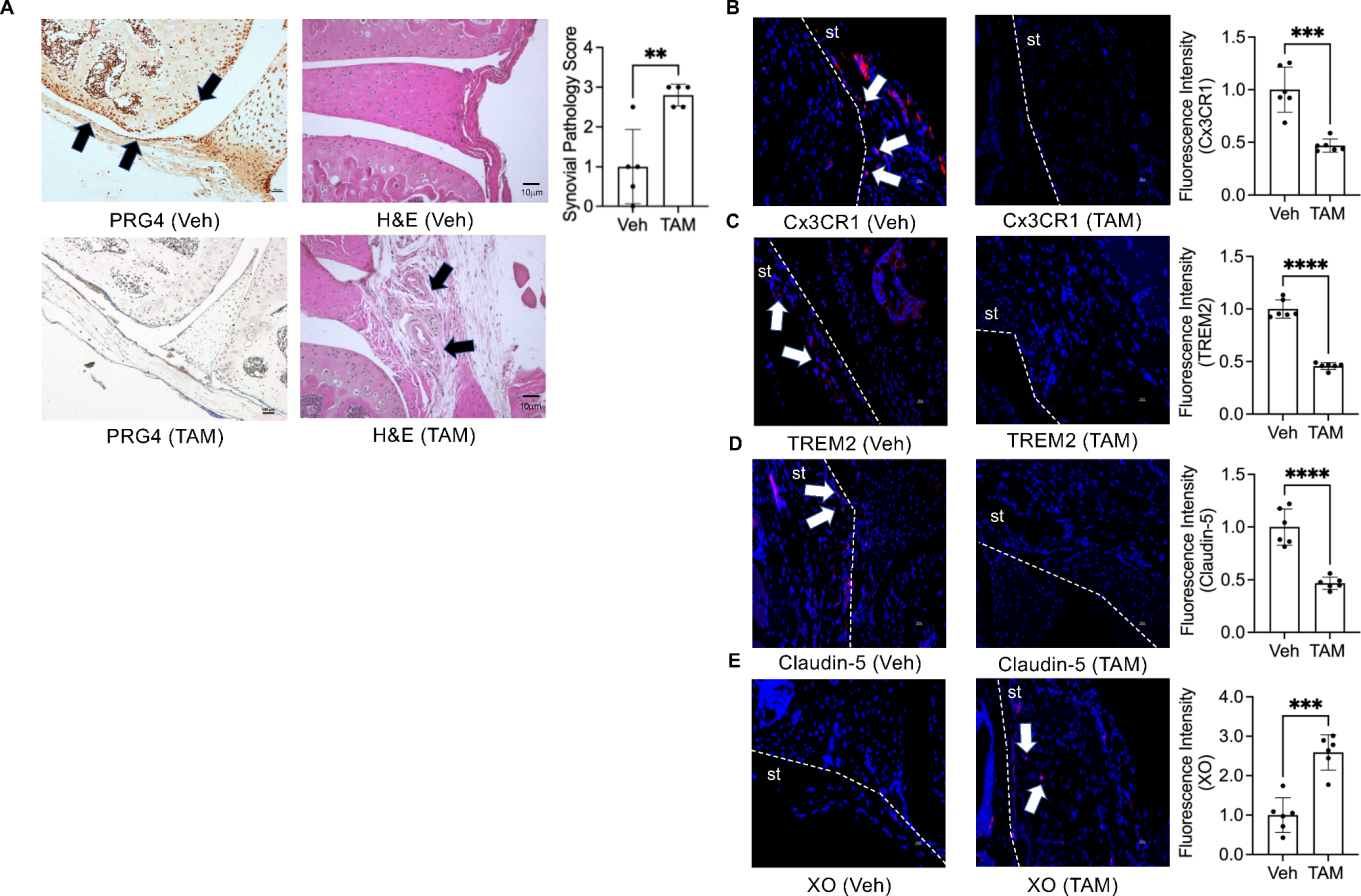
Findings in the synovial lining from proteoglycan 4 (*Prg4*) conditional knockout mice indicate loss of Cx3CR1 + TREM2 + synovial macrophages associated with synovial pathology and induction of xanthine oxidase (XO) expression. (*Prg4*^*FrtloxP/FrtloxP*^;*R26*^*FlpoER/+*^) mice were conditionally inactivated using intraperitoneal tamoxifen (TAM; 0.1 mg/gram) daily for 10 days starting at 4 weeks or corn oil (Veh; 100 μl) and histological analyses were performed 6 weeks later. TAM and Veh groups (*n* = 6 in each group with 3 males and 3 females). ***p < 0.001; ****p < 0.0001. **A.** PRG4 immunostaining is significantly attenuated following TAM administration, associated with increased synovial pathology scores. Arrows point to positive PRG4 staining in Veh animals, and synovial thickening in TAM animals. **B.***Prg4* conditional inactivation reduced Cx3CR1immunostaining in synovial tissues. **C.***Prg4* conditional inactivation reduced TREM2 immunostaining in synovial tissues. **D.***Prg4* conditional inactivation reduced claudin-5 staining in synovial tissues. **E.***Prg4* conditional inactivation increased XO staining in synovial tissues. Arrows point to positive Cx3CR1, TREM2, Claudin-5 and XO staining. Dashed lines were used to demarcate synovial tissues (st)

**Fig. 2:**
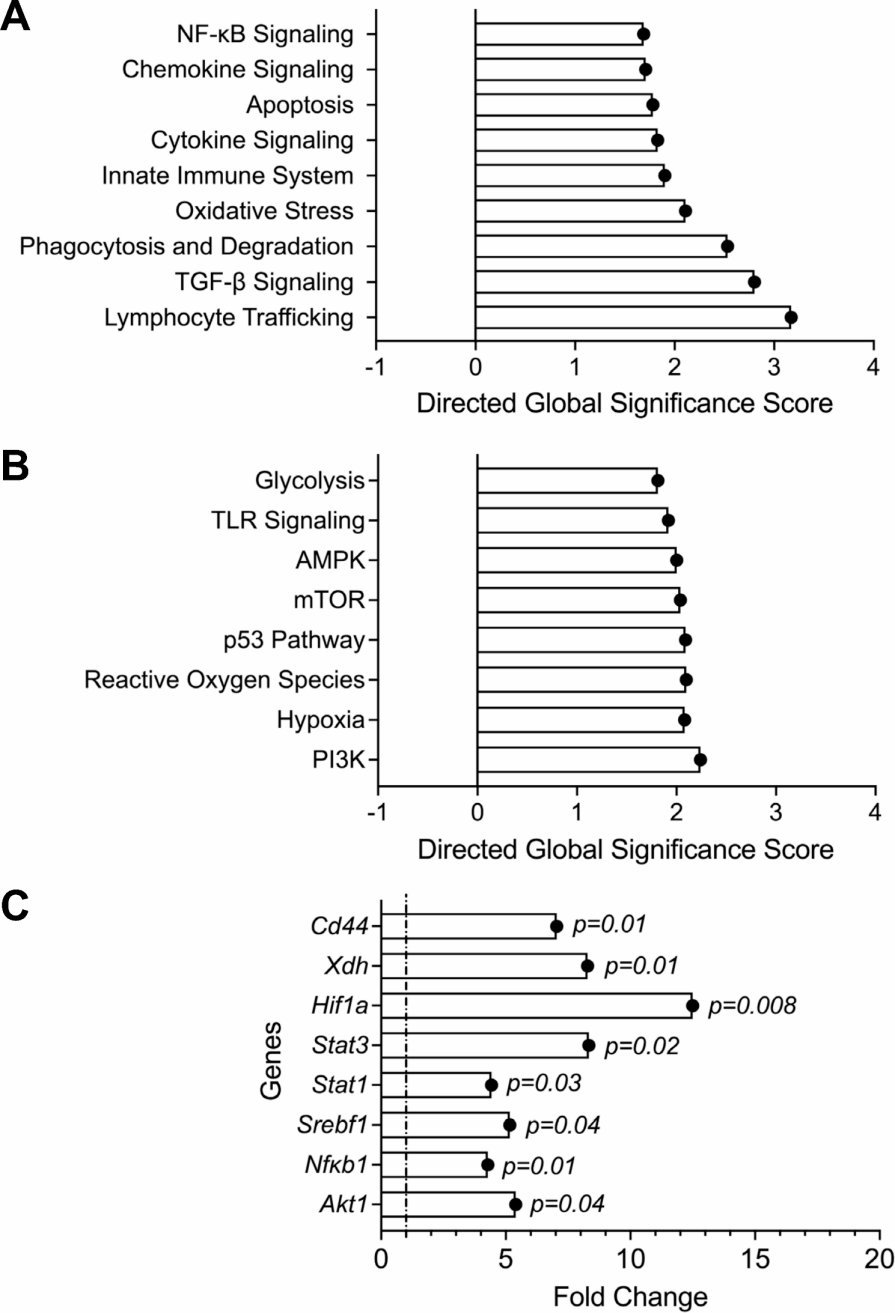
Multiplexed gene expression assays of synovial tissue of *Prg4* conditional knockout mice reveals upregulation of immune and cellular stress pathways. Data is presented as significance scores (pathways) or fold change (individual genes of interest) of TAM vs. Veh (*n* = 6 in each group; 3 males and 3 females). Directed global scores were scaled on the *t*-statistic. Similarly, gene expression data were analyzed using Student’s *t*-test. (**A**) Upregulated immune-related pathways included NF-κB, chemokine and cytokine signaling and oxidative stress. (**B**) Upregulated cell stress pathways included glycolysis, TLR signaling and reactive oxygen species. **C**)*Cd44*, Xanthine oxidoreductase (*Xdh*), hypoxia inducible factor alpha (*Hif1a*) and signature genes of macrophage proinflammatory activation were upregulated in PRG4 deficient synovial tissues

To allow for normal joint development and homeostasis before reducing *Prg4* expression, we utilized a conditional *Prg4* knockout allele (*Prg4*^*FrtloxP/FrtloxP*^;*R26*^*FlpoER/+*^) and Cre-recombinase (*Prg4*^*FrtKO/FrtKO*^) or Flp-recombinase can be used to eliminate PRG4 expression in recombined cells. We assessed the efficiency of Cre recombination following tamoxifen (TAM) administration by droplet-based digital PCR (ddPCR), which allows for quantification of absolute number of *Prg4* copy numbers in recovered synovial tissues from recombined and non-recombined animals (Supplementary Fig. 1). The number of *Prg4* copies in TAM animals (*n* = 3) was 52±9 per 20μL compared to 943±338 in the Veh animals (*n* = 3), approximating 94% reduction in *Prg4* expression. At the protein level, we also observed significant loss of PRG4 staining in articular cells from *Prg4* conditionally inactivated mice (Fig. 1A). The *Prg4* conditionally inactivated synovium displayed significant histopathology (Fig. 1A). We also observed attenuated barrier SM markers, specifically Cx3CR1 (Fig. 1B) and TREM2 (Fig. 1C), along with loss of tight junction protein, claudin-5 (Fig. 1D). The *Prg4* inactivated synovia also expressed XO, which was otherwise absent in wildtype synovia (Fig. 1E). To further elucidate specific pathways that are regulated by PRG4, we conducted multiplexed gene expression studies (Fig. 2) focusing on immunologic and metabolic pathways. We observed upregulation of pathways of innate immunity, cytokine and chemokine signaling and phagocytosis (Fig. 2A). We also observed that oxidative response, glycolysis and hypoxia pathways were activated in *Prg4* inactivated synovia (Fig. 2B). To gain further insight into the activation status of SMs, we analyzed genes that drive macrophage pro-inflammatory activation [35] and discovered that *Hif1a*, *Stat1*, *Stat3*, *Srebf1*, *Nfkb1* and *Akt1* expression levels were higher in *Prg4* inactivated synovia, consistent with a pro-inflammatory macrophage gene signature (Fig. 2C). The PRG4 signaling axis was also disrupted as evidenced by upregulated *Cd44* (∼ 7-fold induction; *p* < 0.01) and *Xdh* (∼ 8-fold induction; *p* < 0.01) expression.

### SMs from Prg4 conditional mice and Prg4 null mice exhibited PRG4 signaling dysfunction and enhanced glycolytic flux

**Fig. 3:**
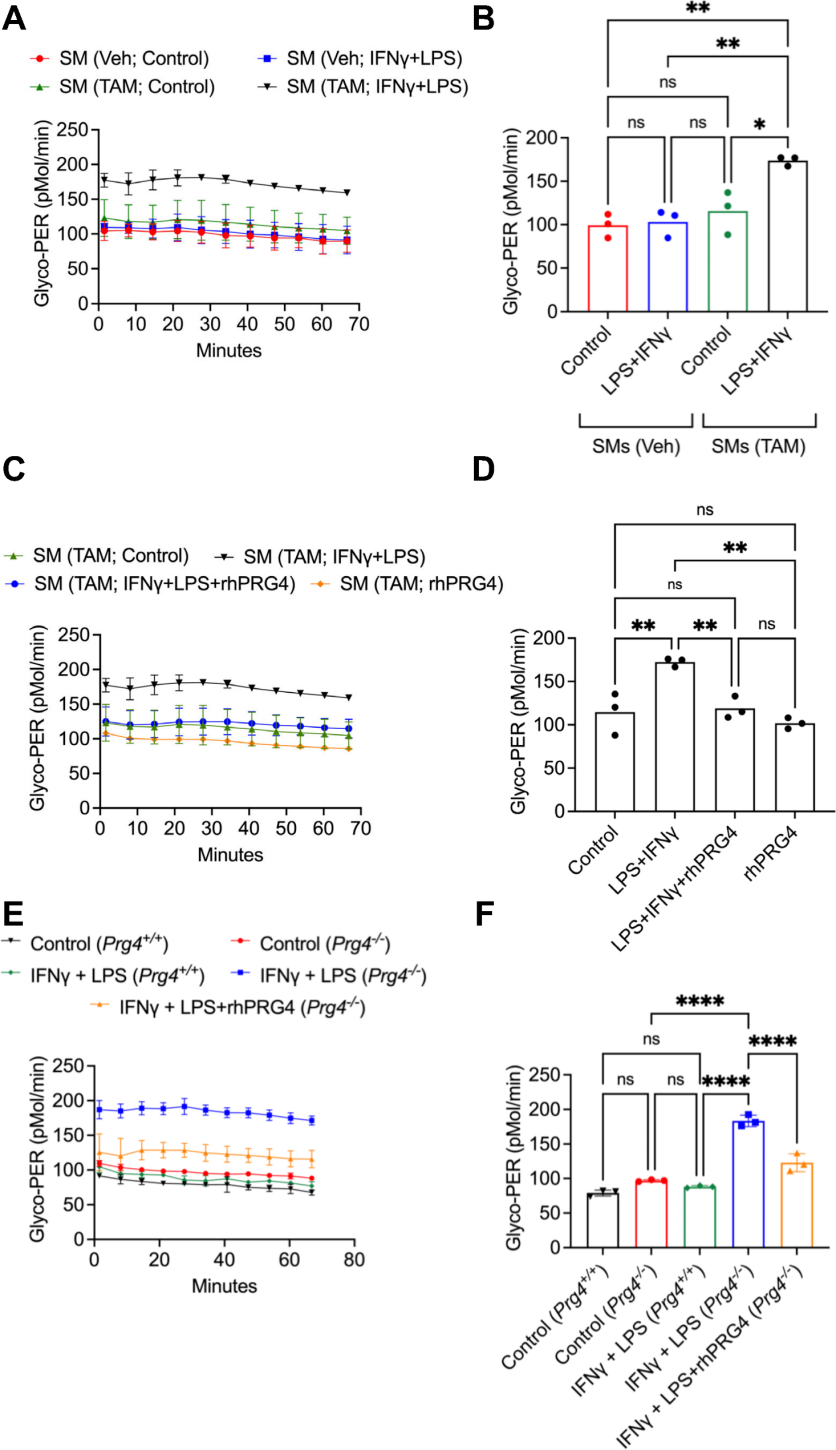
Real-time monitoring of glycolytic activation of cultured synovial macrophages (SMs) from tamoxifen-administered *Prg4* conditionally inactivated (TAM) and vehicle-administered *Prg4* competent (Veh) mice reveals enhanced pro-inflammatory activation of PRG4 deficient SMs which was reversed with recombinant human PRG4 (rhPRG4) (200 μg/ml). Pro-inflammatory activation was achieved by treatment with LPS (100 ng/ml) and IFNγ (20 ng/ml). ns: non-significant; *p < 0.05; **p < 0.01; ***p < 0.001; ****p < 0.0001.**A.** Time-dependent change in glycolytic proton efflux rate (Glyco-PER) in SMs in response to LPS + IFNγ. **B.** SMs from *Prg4* conditionally inactivated mice had higher Glyco-PER values. **C.** Time-dependent change in Glyco-PER in SMs from *Prg4* conditionally inactivated mice in response to rhPRG4. **D.** rhPRG4 reduced pro-inflammatory activation of SMs. **E.** Time-dependent change in Glyco-PER in SMs from *Prg4*^*−/−*^ and *Prg4*^*+/+*^ mice. **F.** rhPRG4 reduced glycolytic activation of SMs from *Prg4*^*−/−*^ animals

**Fig. 4:**
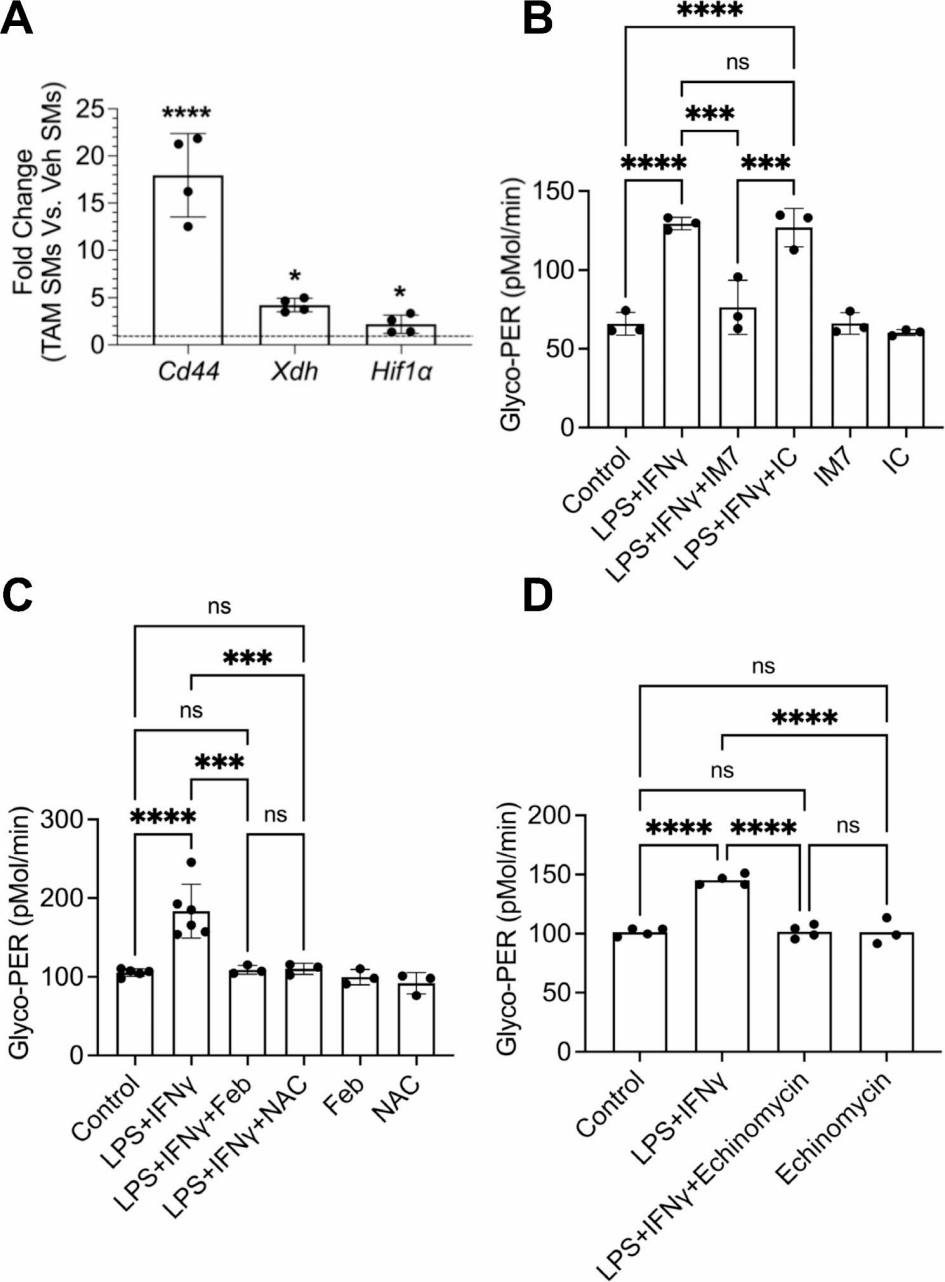
Dysregulation of PRG4 signaling axis in cultured SMs from tamoxifen-administered *Prg4* conditionally inactivated (TAM) mice and reconstitution of PRG4 signaling and its impact on pro-inflammatory activation of these SMs. Target gene expression is presented as fold change in TAM SMs compared to Veh SMs (SMs from vehicle administered *Prg4* competent mice). Pro-inflammatory activation was achieved by treatment with LPS (100 ng/ml) and IFNγ (20ng/ml). ns: non-significant; *p < 0.05; **p < 0.01; ***p < 0.001; ****p < 0.0001.**A.***Cd44*, *Hif1α* and *Xdh* expressions were elevated in SMs from *Prg4* conditionally inactivated mice. **B.** IM7 (15 μg/ml), which induces CD44 extracellular domain cleavage, suppressed pro-inflammatory activation of TAM SMs. **C.** Febuxostat (Feb; 25μM), a xanthine oxidase inhibitor, and NAC (N-acetylcysteine) (10μM), a pan reactive oxygen species (ROS) scavenger, suppressed pro-inflammatory activation of TAM SMs. **D.** Echinomycin (50nM), a HIF-1α inhibitor, suppressed pro-inflammatory activation of TAM SMs

We used glycolytic activation of cultured SMs from *Prg4* inactivated and vehicle control mice to decipher the mechanistic pathway by which PRG4 modulates SM activation. SMs from *Prg4* conditionally inactivated mice were readily activated by LPS + IFNγ compared to SMs from control counterparts (*p < 0.01*; Fig. 3A and B). Glycolytic activation of LPS + IFNγ treated SMs was reversed by addition of rhPRG4 (*p < 0.01*) (Fig. 3C and D). This finding was also reproduced in SMs from *Prg4* knockout mice (*Prg4*^*−/−*^) and their *Prg4*^*+/+*^ control counterparts (Fig. 3E and F). Glycolytic switching in SMs from *Prg4*^*−/−*^ mice was observed following LPS + IFNg stimulation (*p < 0.0001*) and rhPRG4 protected SMs from activation (*p < 0.0001*). The concordance of evidence derived from two independent *Prg4* genetic models highlights the importance of PRG4 signaling in SMs in the context of their pro-inflammatory activation. We subsequently examined the dysregulation of PRG4 signaling at the gene level in *Prg4* conditionally inactivated SMs and investigated whether reconstitution of PRG4 signaling suppressed glycolytic activation. *Cd44* (∼ 18 fold) (*p < 0.0001*), *Xdh* (∼ 4 fold) (*p < 0.05*), and *Hif1a* (∼ 2 fold) (*p < 0.05*) expressions were increased in *Prg4* conditionally inactivated SMs (Fig. 4A). IM7, which induces CD44 extracellular domain shedding [36], suppressed glycolytic activation of *Prg4* conditionally inactivated SMs compared to isotype control (*p < 0.001*; Fig. 4B), and XO inhibition by febuxostat (*p < 0.001*; Fig. 4C) yielded similar results. ROS appeared to be causally implicated in glycolytic activation of these SMs as a pan-ROS inhibitor, N-acetylcysteine (NAC), mimicked the effect of febuxostat (*p < 0.001*; Fig. 4C). HIF-1α appeared to also play a causal role in activation of SMs as echinomycin, a HIF-1α inhibitor [37], treatment suppressed glycolytic activation of these cells (*p < 0.0001*; Fig. 4D). 

### XO-generated ROS increases HIF-1α in activated SMs

**Fig. 5:**
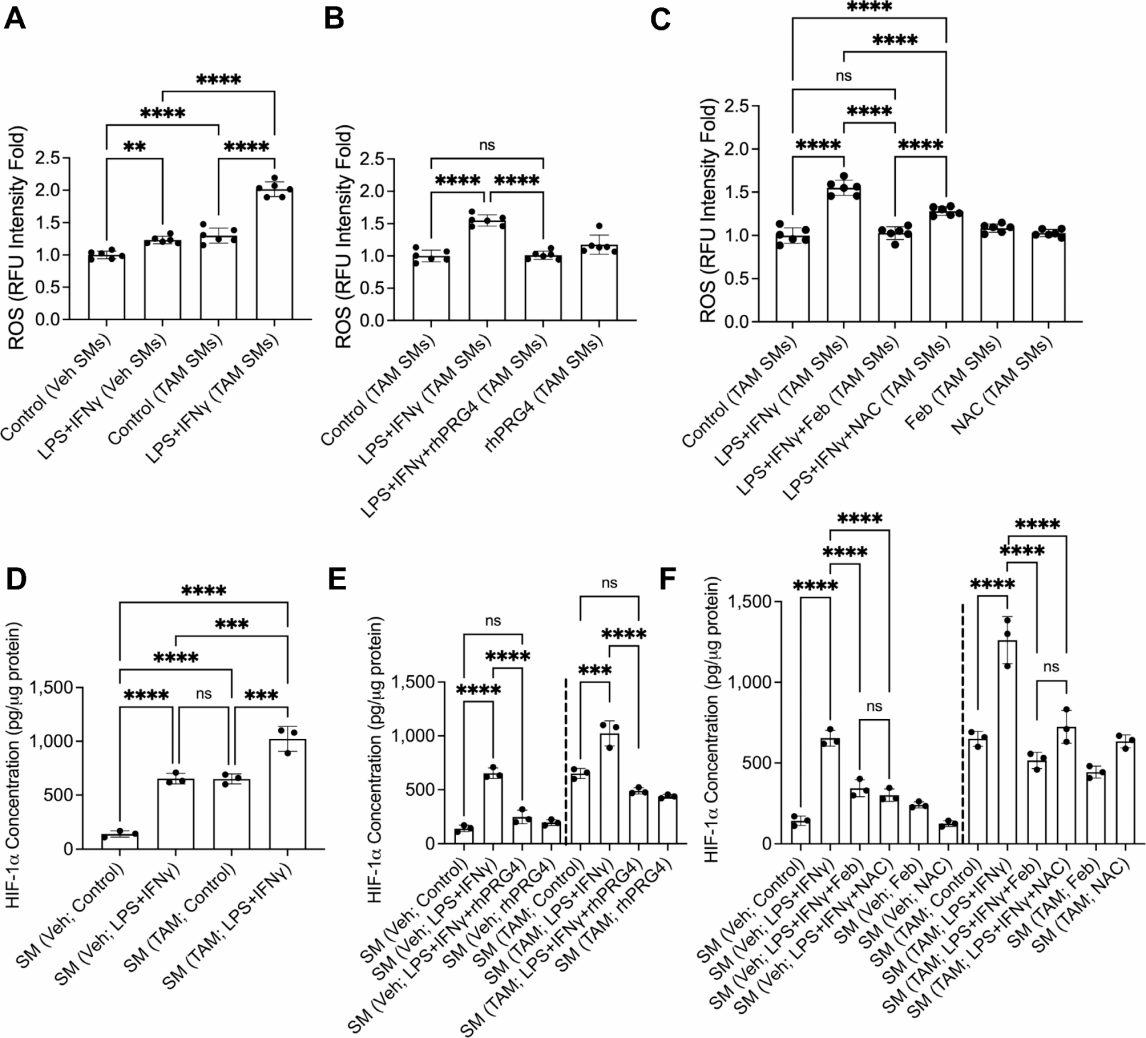
Reactive oxygen species (ROS) generation by cultured SMs from *Prg4* conditional knockout mice and hypoxia inducible factor alpha (HIF-1a) induction are reversible with XO inhibition. SMs from tamoxifen (TAM) or vehicle (Veh) administered mice were isolated and stimulated with LPS (100ng/ml) and IFNγ (20ng/ml) and ROS was determined fluorometrically. HIF-1α levels were measured by ELISA. ns: non-significant; *p < 0.05; **p < 0.01; ***p < 0.001; ****p < 0.0001.**A.** ROS generation was higher in stimulated TAM SMs. **B.** Recombinant human PRG4 (rhPRG4; 200 μg/ml) reduced ROS generation in response to LPS + IFNγ. **C.** Febuxostat (Feb; 25μM) and NAC (10μM) reduced ROS generation in response to LPS + IFNγ. **D.** HIF-1α levels in TAM SMs were higher at baseline and in response to LPS + IFNγ. **E.** Recombinant human PRG4 (rhPRG4; 200 μg/ml) reduced HIF-1α levels in response to LPS + IFNγ. **F.** Febuxostat (Feb; 25μM) and NAC (10μM) reduced HIF-1α levels in response to LPS + IFNγ

We studied ROS generation in SMs from *Prg4* conditionally inactivated mice and identified that ROS levels in these cells were higher than control counterparts at baseline and in response to LPS + IFNγ (*p < 0.0001* for both comparisons; Fig. 5A). rhPRG4 (*p < 0.0001*; Fig. 5B) reduced ROS levels in SMs, as did direct inhibition of their generation by XO (*p < 0.0001*; Fig. 5C). Elevated HIF-1α levels were also observed in SMs from *Prg4* conditionally inactivated mice (*p < 0.0001*), and HIF-1α was induced in these SMs in response to LPS + IFNγ (*p < 0.001*) (Fig. 5D). rhPRG4 corrected HIF-1α levels (*p < 0.0001*; Fig. 5E), as did febuxostat (*p < 0.0001*; Fig. 5F). Taken together, these data suggest that activation of PRG4 signaling downregulated HIF-1α levels, mediated by suppressing ROS generation by XO. By contrast, induction of HIF-1α promoted glycolysis and SM activation.

### Oral febuxostat reduced systemic inflammation, promoted barrier SMs and reduced synovitis in vivo

**Fig. 6:**
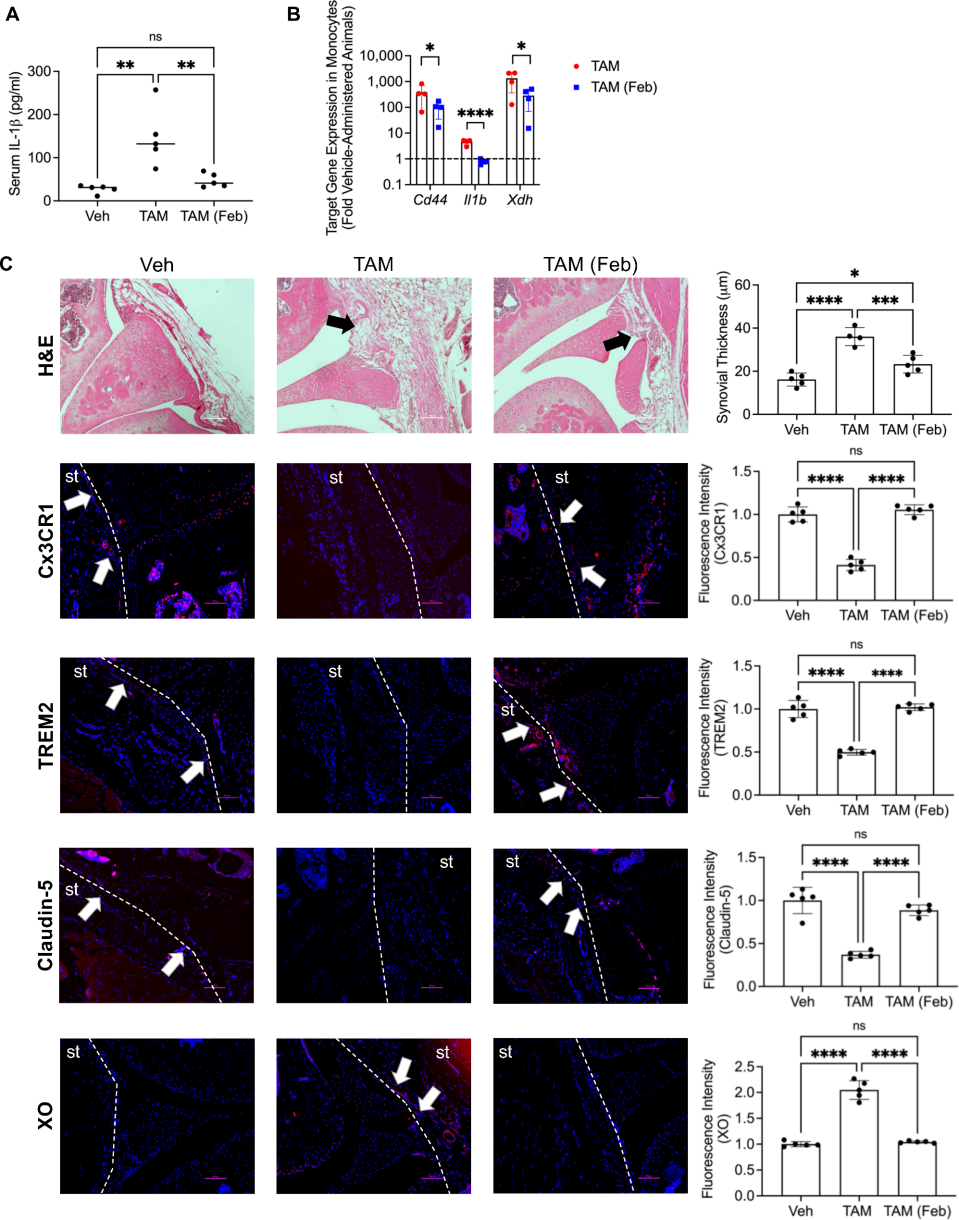
Oral febuxostat treatment corrects systemic inflammation and normalizes synovial pathology in proteoglycan 4 (*Prg4*) conditional knockout mice. Tamoxifen-administered Prg4 deficient mice (TAM) were treated with oral febuxostat (Feb; 2.5 mg/kg/day in drinking water) or placebo for 6 weeks (*n* = 5 in each group; 3 males and 2 females). Analyses included serum interleukin-1 beta (IL-1β) levels by ELISA and qPCR of target genes in isolated peripheral monocytes. Histological analyses included hematoxylin and eosin (H&E), immunostaining of Cx3CR1, TREM2, Claudin-5 and xanthine oxidase (XO). Vehicle (Veh) administered *Prg4* competent mice (*n* = 5; 3 males and 2 females) were used as controls. ns: non-significant; *p < 0.05; **p < 0.01; ***p < 0.001; ****p < 0.0001. (**A**) Oral febuxostat reduced serum IL-1β levels in *Prg4* conditionally inactivated mice. (**B**) Oral febuxostat reduced *Cd44*, *Il1b* and *Xdh gene* expression in peripheral monocytes from *Prg4* conditionally inactivated mice. (**C**) Oral febuxostat treatment reduced synovial thickness, enhanced Cx3CR1, TREM2 and Claudin-5 while simultaneously reducing XO synovial staining in *Prg4* conditionally inactivated mice. Dashed lines were used to demarcate synovial tissues (st)

We also investigated whether XO inhibition attenuated inflammation and corrected synovial pathology in *Prg4* conditionally inactivated mice. Systemic IL-1β levels were elevated in *Prg4* inactivated mice (*p < 0.01*; Fig. 6A), and febuxostat treatment was anti-inflammatory, as it reduced circulating IL-1β levels (*p < 0.01*; Fig. 6A). In circulating monocytes, the anti-inflammatory effect of febuxostat was also observed as expression of *Cd44* (*p < 0.05*), *Il1b* (*p < 0.0001*) and *Xdh* (*p < 0.05*) were reduced following treatment (Fig. 6B). In the synovium, febuxostat reduced synovial thickness (*p < 0.001*), enhanced Cx3CR1 (*p < 0.0001*), TREM2 (*p < 0.0001*) and claudin-5 (*p < 0.0001*), while simultaneously reducing XO (*p < 0.0001*) staining (Fig. 6C). Our in vivo observations suggest that systemic inflammation in the *Prg4* conditional mouse was reversible and that correcting PRG4 signaling dysfunction, *via* XO inhibition, promoted the resolution of inflammation in the synovium and the re-establishment of the barrier SM layer.

### rhPRG4 reduced glycolytic activation of CD14 + monocytes/macrophages from OA synovial tissues

Medial compartment CD14 + monocytes/macrophages had higher glyco-PER compared to lateral compartment counterparts (*p < 0.0001*) (Supplementary Fig. 2A). rhPRG4 treatment reduced glycolytic activation of CD14 + cells from the medial compartment (Supplementary Fig. 2B) and lateral compartment (Supplementary Fig. 2C) (*p < 0.0001* for both comparisons).

## Discussion

In this work, we investigated PRG4’s role in maintaining synovial architecture with a particular focus on its SM homeostatic function (Supplementary Fig. 3). PRG4 protects the SM barrier layer and prevents the activation of interstitial macrophages, mediated by XO induction which in turn drives synovitis. XO inhibition suppressed SM activation by blocking XO-generated ROS and HIF-1α induction and, in vivo, corrected synovial pathology and promoted the reconstitution of the SM barrier. More importantly, we showed that a clinically available drug can be successfully repurposed to ameliorate synovitis in a pre-clinical model of CACP, which also provides the rationale for its use in other chronic joint diseases highlighted by chronic synovitis.

A highlight of our findings was that PRG4 appears to protect the immunological barrier in the synovial lining. A combination of loss of the anti-inflammatory Cx3CR1 + TREM2 + lining macrophages and tight junction protein claudin-5 was potentially consequential to the activation of interstitial macrophages. Indeed, we observed an associated upregulation of XO expression in the synovium, detected at the gene and protein levels, as well as an upregulated gene signature for *pro*-inflammatory macrophages [35]. NF-κB, STAT1, STAT3, and HIF-1α gene expressions were at least 4-fold upregulated in synovial tissues of *Prg4* conditional knockout mice. These transcription factors are known to induce the expression of pro-inflammatory macrophage markers CD80, CD86, iNOS, COX2, MHC-II and release of pro-inflammatory cytokines [38, 39]. The multiplexed gene expression studies further revealed that with PRG4 loss, there is a robust activation of innate immune and metabolic stress responses by the synovium. Glycolysis and oxidative stress are two novel pathways that were revealed to be upregulated in synovial tissues of the *Prg4* conditional knockout mouse in its *Prg4* null state. Synovial pathology was also evident in the new mouse, which was also consistently observed in other *Prg4* mutant mouse models [8, 10].

We performed follow-up experiments to differentiate glycolysis and oxidative stress pathways in isolated synovial macrophages. Enhanced glycolytic flux and accumulation of ROS in response to LPS + IFN were detected in SMs harvested from *Prg4* conditionally inactivated synovial tissues. The loss of PRG4 induced resident SMs into a metabolic state that predominantly relied on glycolysis which is essential in transitioning to pro-inflammatory macrophages [35]. In contrast, SMs isolated from PRG4 competent controls displayed minimal pro-inflammatory activation under the same conditions. The glycolytic switch was also observed in SMs from animals born lacking germline *Prg4* expression. Correlated to this, we have previously reported that in a *Prg4* gene-trap mouse model, animals born lacking PRG4 displayed progressive loss of Cx3CR1 + synovium-resident macrophages, and accumulation of CD86 + pro-inflammatory macrophages which was reversed with *Prg4* re-expression [32]. In SMs, CD44 upregulation appeared to play a key role in glycolytic pro-inflammatory activation as CD44 extracellular matrix shedding reduced glycolysis (Fig. 4) and ROS accumulation in these cells (Fig. 5). Our observation is in agreement with previous reports which showed that CD44 ablation resulted in a glycolysis-to-oxidative phosphorylation transition [40]. In this context, we can understand the role that PRG4 plays in suppressing SM activation via glycolysis and ROS accumulation due to its interaction with CD44 and the internalization of PRG4-CD44 [9] which reduces the unoccupied CD44 density on macrophages.

HIF-1α is a key modulator of glucose metabolism in cells, as an adaptive mechanism to promote cell survival [41]. HIF-1α was shown to upregulate genes of glucose transporters and enzymes of the glycolytic pathway, while suppressing oxidative phosphorylation [41]. Cellular oxygen tension is a major regulator of HIF-1α as adequate cellular oxygen levels induce HIF-1α hydroxylation resulting in a short half-life, estimated to be 5–10 min [41]. In contrast and under hypoxic conditions, HIF-1α is protected from hydroxylation and is therefore stabilized, resulting in an increase in its cellular levels [41]. ROS was shown to contribute to HIF-1α stabilization via a multi-pronged approach including depleting the substrate of prolyl hydroxylase, responsible for HIF-1α hydroxylation, and inhibition of factor inhibiting HIF [42]. In our work, HIF-1α appeared instrumental in mediating the observed glycolytic flux in synovial macrophages from *Prg4* null mice. HIF-1α levels were elevated in these macrophages at both the transcriptional and protein levels and activation of the PRG4 signaling axis reduced cellular HIF-1α. Furthermore, a direct HIF-1α inhibitor treatment produced a similar effect to that observed with PRG4 signal axis activation. These observations collectively point to HIF-1α as a downstream effector of PRG4 dysfunction in SMs. Of interest, and aligning with our findings, Ruan et al. have shown that PRG4 downregulated HIF-1a expression in cartilage as a mechanism of inhibiting its turnover in OA [43].

To further evaluate the role of ROS in regulating the glycolytic flux in isolated SMs, we initially determined the extent of ROS accumulation in SMs from *Prg4* inactivated mice and found that at baseline, ROS level was ∼ 30% higher than corresponding level from *Prg4* competent mice, and a pro-inflammatory stimulus increased ROS levels by approximately 2-fold. Major sources of cytosolic ROS include NADPH oxidase and XO [42]. In *Prg4* conditionally null SMs, XO gene expression was induced by ∼ 5-fold (Fig. 4) and XO inhibition suppressed basal and LPS induced ROS accumulation. The downstream response to ROS depletion was a reduced glycolytic flux and HIF-1α levels (Fig. 6), likely due to ROS interference with HIF-1α hydroxylation-mediated stabilization. The posited role of ROS in activating SMs that have PRG4 signaling dysfunction is not exclusive of enhanced glycolytic flux. ROS was shown to activate the NF-κB and the Jak/STAT pathways [42]. In our study, the NF-κB pathway was upregulated in *Prg4* null synovial macrophages as did the expression of transcription factors STAT1 and STAT3. Furthermore, ROS directly inactivates the PRG4 signaling axis, thereby creating a positive feedback loop that augments the activation of macrophages [44]. Our results with NAC further confirmed the implication of ROS in the activation of SMs since NAC reproduced similar in vitro effects to those of febuxostat. Taken together, our data support that PRG4 signaling axis dysfunction, mediated by PRG4 loss, induced XO expression and increased ROS accumulation in SMs. XO-generated ROS inhibited HIF-1α degradation and thus elevated HIF-1α cytosolic levels. HIF-1α orchestrated a metabolic shift towards glycolysis which was permissive for pro-inflammatory activation. Furthermore, it is evident that restoring PRG4 signaling, via re-introduction of rhPRG4, inducing CD44 shedding, or suppressing XO activity reduced glycolytic flux and pro-inflammatory activation mediated by suppressing ROS and HIF-1α degradation.

Macroscopic evidence of synovitis is a common finding in up to 74% of patients with knee OA of different grades and in 95% of patients with moderate to severe OA [45]. Baseline severity of synovitis is also associated with radiographic worsening of knee OA, more cartilage damage and erosions in hand OA [45]. Synovial fibrosis, common in advanced OA, is an independent contributor to joint pain and stiffness [46]. We have previously shown that fibrosis is a key component of synovial tissue remodeling in a *Prg4* gene trap mouse model and is reversible upon *Prg4* re-expression [20]. In the present work, we sought to investigate whether reconstituting PRG4 signaling, by febuxostat, reversed synovial pathology and re-established the SM barrier. We treated our *Prg4* conditionally inactivated mice with oral febuxostat, at a dose that reduced systemic inflammation in mice [47] and used serum IL-1β as a marker of systemic inflammation. Oral febuxostat reduced serum IL-1β levels and attenuated monocytes’ *Il1b*, *Xdh* and *Cd44* expressions. This effect points to the efficacy of febuxostat as an anti-inflammatory agent which reduced monocyte activation in our animal model. A lower CD44 surface density on monocytes may reduce the likelihood of their ingress into the synovium since CD44 is required for classical monocyte extravasation and differentiation. This is supported by our previous observation that the synovial ingress of Cx3CR1^lo^ CCR2^hi^ XO^hi^ classical monocytes was elevated in *Prg4* null mice [48]. In synovial tissues, febuxostat treatment reversed synovial hyperplasia, promoted the expression of tight junction protein claudin-5, enriched the synovium with Cx3CR1 + TREM2 + SMs and attenuated XO expression by interstitial macrophages since Type B synoviocytes do not express XO [27]. Having established the anti-inflammatory efficacy of febuxostat in conditionally null *Prg4* mice is a novel finding that may be a translatable finding for patients with CACP [11]. This will constitute a novel repurposing of febuxostat, which is clinically indicated to lower serum uric acid level in patients with gout, where it was shown to reduce synovial inflammation [49]. Our studies on human CD14 + monocytes further confirm the glycolytic activation of CD14 + cells from the medial compartment is reversible with rhPRG4, an effect that may contribute to rhPRG4’s observed therapeutic efficacy in post-traumatic OA [3].

In summary, we utilized a novel mutant mouse model where post-natal *Prg4* gene inactivation allowed us to capture, in real-time, early metabolic changes in SMs and associated changes in synovial tissue architecture. We identified that PRG4 signaling dysfunction in SMs facilitated their enhanced glycolytic flux that is required for pro-inflammatory activation with XO-derived ROS fulfilling an effector function that regulates HIF-1α activity. Structurally, the immunological barrier in the synovium was disrupted in the absence of PRG4 and this was permissive for activation of immune and cell stress response pathways that facilitated chronic synovitis. Interestingly the interplay between PRG4 and claudin-5 in a structural barrier was also observed in the blood brain barrier of a rat traumatic brain injury model [50].

### Limitations

One limitation of our work was that we did not examine the synovial reactivity to an acute traumatic joint insult or a gout flare, overlayed on chronic inflammation in our model. We focused our studies on the synovium, given the emerging role of PRG4 as an immunomodulator in the joint. We examined changes to the immune environment in the synovium using one end point (6 weeks) following *Prg4* inactivation. In the future, we plan to study changes in the synovial immune environment at later time points (6 months) to assess long-term impact of PRG4 loss on synovial immune reactivity. We performed glycolytic assays using SMs that potentially included both resident and monocyte-derived populations. We can infer that these SMs, on average, underwent an enhanced glycolytic shift in response to a pro-inflammatory signal. However, we are not able to dissect the glycolytic shift of resident SMs vs. monocyte-derived SMs.

## Conclusions

PRG4 plays a key role in promoting SM homeostasis. Failure of PRG4’s biological role predisposes the synovium to long-term pathological changes, which are reversible with direct XO inhibition.

## Electronic supplementary material

Below is the link to the electronic supplementary material.

Supplementary Material 1

## Data Availability

Data is provided within the manuscript and is available upon request.

## Abbreviations

CACP: Camptodactyly-Arthropathy-Coxa Vara Pericarditis
CD44: Cluster of differentiation 44
COX2: Cyclo-oxygenase 2
HIF-1α: Hypoxia inducible factor alpha
IFN-γ: Interferon gamma
IL-1β: Interleukin 1 beta
iNOS: Inducible nitric oxide synthase
LPS: Lipopolysaccharide
MHC-II: Major histocompatibility complex II
NF-κB: Nuclear factor kappa B
OA: Osteoarthritis
PRG4: Proteoglycan 4
ROS: Reactive oxygen species
SM: Synovial Macrophage
TLR: Toll-like receptor
XO: Xanthine oxidase
