# Supplementary material for: Proteoglycan 4 (Lubricin) and regulation of xanthine oxidase in synovial macrophage as a mechanism of controlling synovitis
Source: Arthritis Res Ther. 2024 Dec 19;26:214. doi: 10.1186/s13075-024-03455-x (PMC11657766; doi:10.1186/s13075-024-03455-x)
Supplement: Supplementary file 1 — Supplementary Material 1 [file 13075_2024_3455_MOESM1_ESM.docx]

**
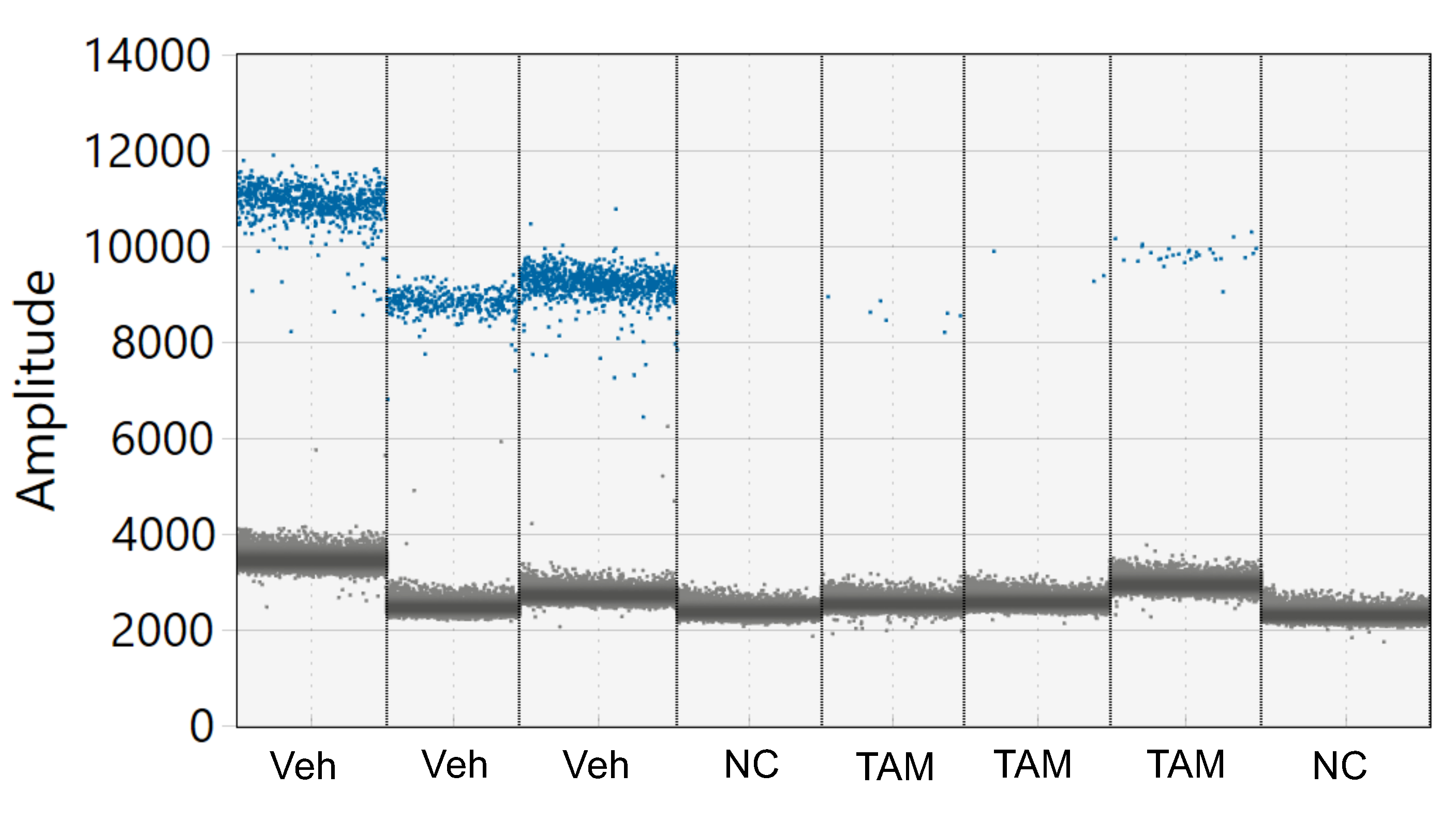
**

**Supplementary Figure 1** Efficiency of *Prg4* recombination in synovial tissues from vehicle (Veh) and tamoxifen (TAM) administered animals. *Prg4* levels were determined by droplet digital polymerase chain reaction (ddPCR) on cDNA from Veh and TAM synovial tissues (n=3 in each group at 6 weeks following recombination). *Prg4* levels were reduced in TAM animals (blue dots represent positive droplets and black dots represent negative droplets). NC=Negative Control.


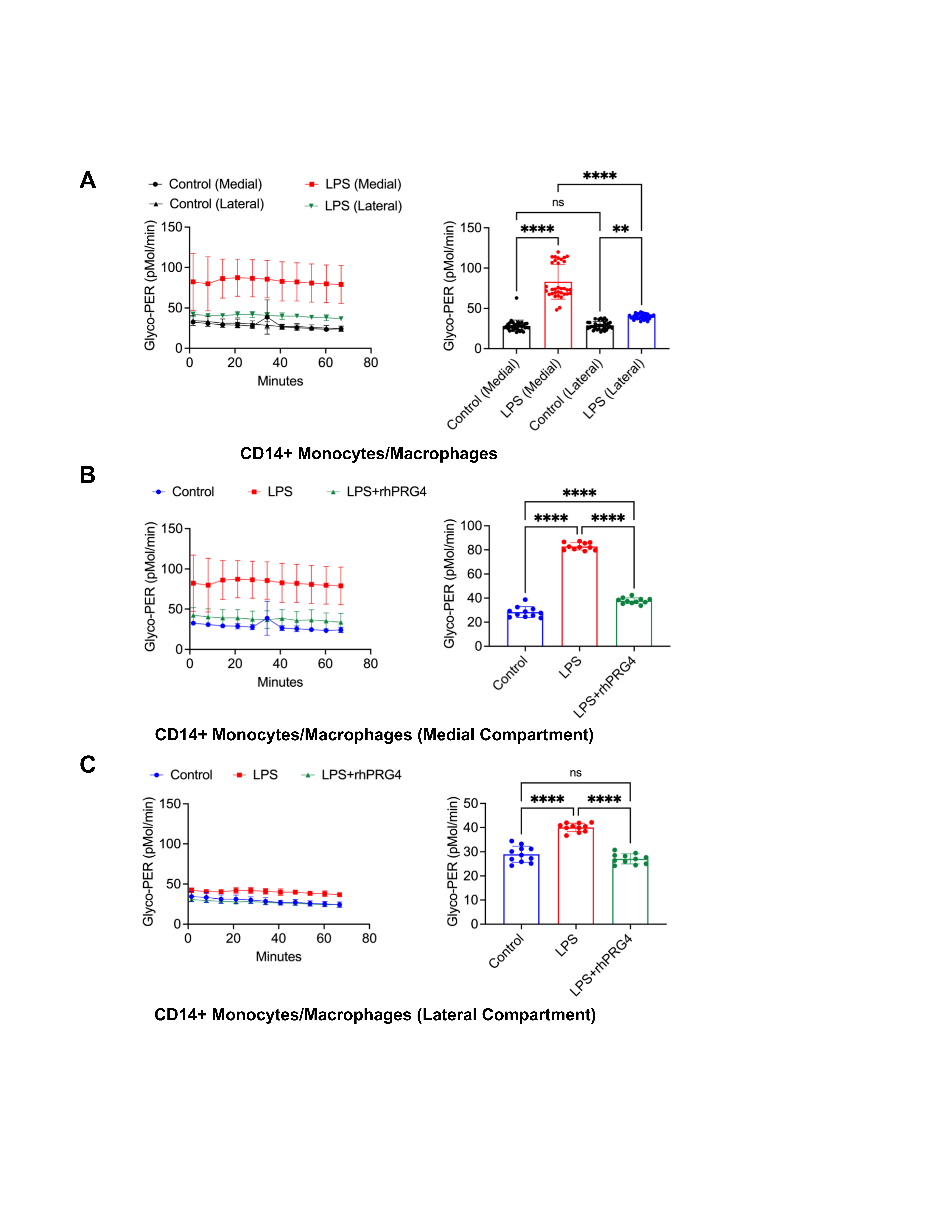


**Supplementary Figure 2** Glycolytic activation of human CD14+ monocytes/macrophages isolated from synovial tissues (medial and lateral compartments) from three end-stage osteoarthritic patients undergoing knee arthroplasty. ns: non-significant; ***p<0.01*; *****p<0.0001*. **A.** Medial compartment CD14+ monocytes/macrophages were activated by LPS to a higher extent than lateral compartment CD14+ monocyte/macrophages. **B.** rhPRG4 treatment reduced medial compartment CD14+ monocyte/macrophage glycolytic activation. **C.** rhPRG4 treatment reduced lateral compartment CD14+ monocyte/macrophage glycolytic activation.


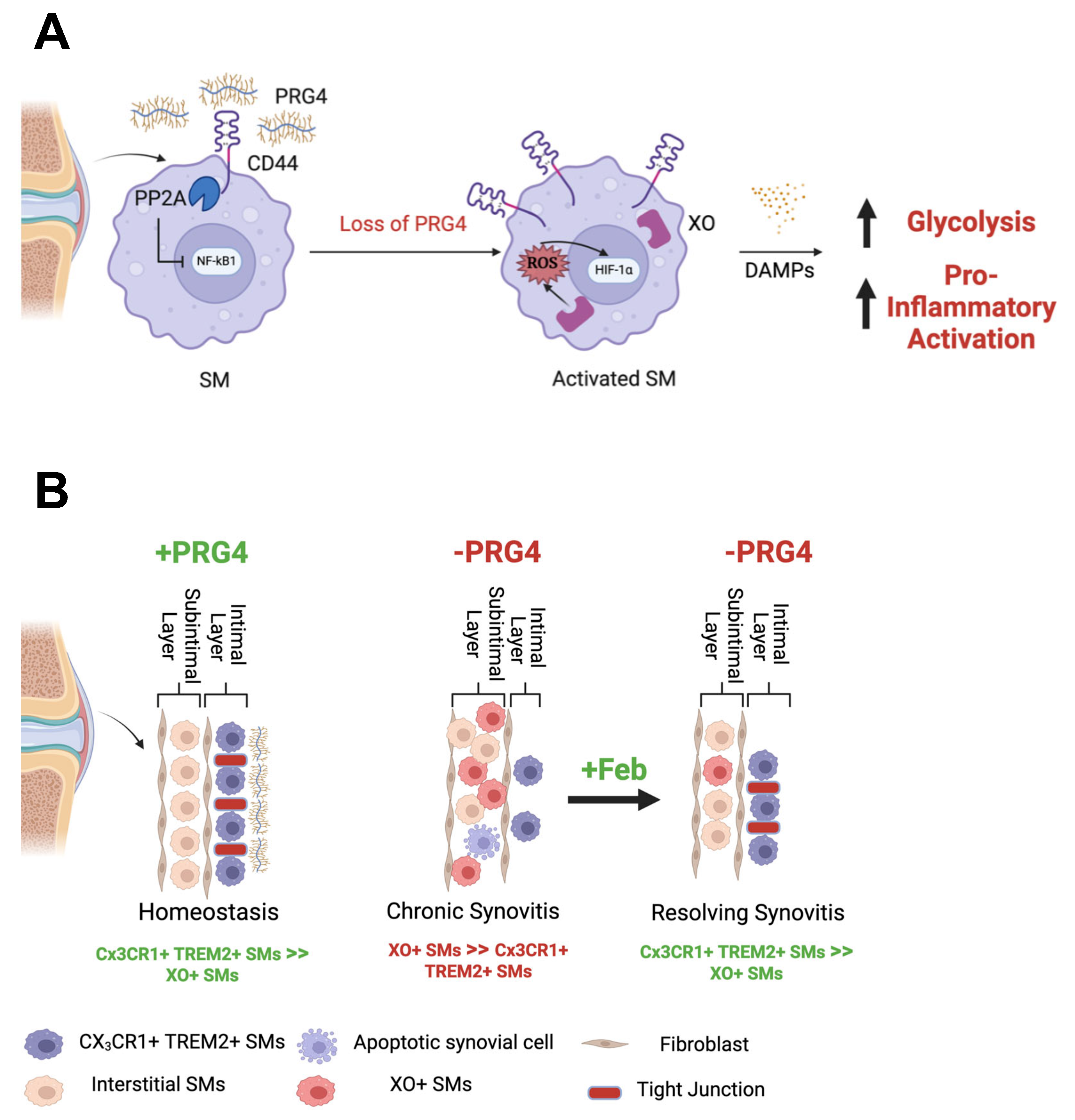


**Supplementary Figure 3** Graphic depicting the identified role of PRG4 in the synovium in the context of balance between anti-inflammatory Cx3CR1+ TREM2+ synovial macrophages (SMs) and pro-inflammatory xanthine oxidase (XO)+ SMs. **A.** Loss of synovial PRG4 upregulates CD44 expression on SMs and induces XO. Reactive oxygen species (ROS) generated by XO activates hypoxia inducible factor alpha (HIF-1a) and mediates glycolysis and pro-inflammatory activation. **B.** Loss of PRG4 depletes the synovium of Cx3CR1+ TREM2+ SMs and increases XO+ SMs and enrichment of Cx3CR1+ TREM2+ SMs and depletion of XO+ SMs in the synovium is accomplished with febuxostat (Feb) treatment.
